# Human herpesvirus 6B U65 binds to histone proteins and suppresses interferon production

**DOI:** 10.1128/jvi.00984-25

**Published:** 2025-09-15

**Authors:** Haokun Li, Hirohito Ogawa, Da Teng, Yuki Okame, Hikaru Namba, Tomoyuki Honda

**Affiliations:** 1 Department of Virology, Okayama University Graduate School of Medicine, Dentistry and Pharmaceutical Sciences 199491 , Okayama, Japan; 2 Department of Virology, Faculty of Medicine, Dentistry and Pharmaceutical Sciences, Okayama University 12997, Okayama, Japan; 3 Faculty of Veterinary Medicine, Okayama University of Science 724947 , Imabari City, Ehime, Japan; University of Virginia, Charlottesville, Virginia, USA

**Keywords:** HHV-6B, interferons, histone, tegument, U65

## Abstract

Human herpesvirus 6B (HHV-6B), a member of the *Betaherpesvirinae* subfamily, is a T-lymphotropic virus that causes exanthem subitum and has been implicated in neuroinflammatory conditions such as multiple sclerosis. The tegument proteins, which are characteristic of herpesviruses, play a crucial role in the envelopment of virions and evasion of host immune defenses, such as the interferon β (IFNβ) signaling pathway. However, the precise mechanisms through which the HHV-6B tegument proteins modulate the IFNβ pathway are not yet fully understood. In this study, we identified a novel function of the HHV-6B tegument protein U65 as an inhibitor of IFNβ production. Additionally, two host histone proteins, hCG_2039566 (H2ACG) and H2AC7, were identified as positive regulators of innate immune pathways. U65 interacts with H2ACG and H2AC7, impairing their ability to promote the IFNβ pathway. Furthermore, we demonstrated that U65 plays critical roles during HHV-6B infection. This study highlights a critical strategy employed by HHV-6B to evade immune defenses, shedding light on its mechanisms for counteracting host responses.

**IMPORTANCE:**

HHV-6B is a virus that primarily infects T cells and can cause illnesses like exanthem subitum and is linked to neurological conditions such as multiple sclerosis. Like other herpesviruses, HHV-6B likely has special proteins that help it avoid the body’s immune defenses, such as the IFNβ signaling pathway, which plays a key role in fighting viral infections. However, how these viral proteins interfere with the immune response is not yet fully understood. In this study, we discovered that one of the HHV-6B proteins, U65, blocks the production of IFNβ, weakening the antiviral defenses. We also found that two human proteins, hCG_2039566 and H2AC7, promote the immune system, but U65 prevents them from doing so. This study reveals a new way that HHV-6B escapes the immune system, providing insight into how the virus efficiently establishes infections and how we might target its ability to evade immunity.

## INTRODUCTION

Human herpesvirus 6B (HHV-6B) is a widely distributed virus classified under the *Roseolovirus* genus of the *Betaherpesvirinae* subfamily. It is a T-lymphotropic herpesvirus that causes exanthem subitum (1–4). Although HHV-6B is also identified in patients with neuroinflammatory conditions such as multiple sclerosis, its causal association with these conditions remains uncertain (5, 6). Herpesviruses are characterized by the capsid containing double-stranded DNA (dsDNA), the envelope, and the tegument (7). Among them, the tegument is a structural hallmark unique to herpesviruses. The tegument consists of multiple tegument proteins critical for herpesvirus replication. To date, 17 tegument proteins have been identified in HHV-6B, with studies indicating that these proteins may play an important role in HHV-6B replication and pathogenesis (8–10). However, their precise mechanisms of action remain incompletely understood.

Interferons (IFNs) are antiviral cytokines crucial for the innate immune response to viral infections. In response to viral infection, cells produce and release type I IFNs that act on themselves and neighboring cells, triggering the transcription of hundreds of IFN-stimulated genes (ISGs). These gene products combat viral infections directly, e.g., by inhibiting viral replication, or indirectly by modulating subsequent immune responses (11, 12). Among the various subtypes of type I IFNs, IFNβ can be produced by almost all cells in the body (12, 13). During infection, dsDNA of herpesviruses is detected by pattern recognition receptors (PRRs), such as cyclic GMP–AMP synthase (cGAS). The binding of cGAS to viral dsDNA allosterically activates its catalytic activity and leads to the production of 2′3′-cyclic GMP–AMP (cGAMP), a second messenger molecule that stimulates stimulator of interferon genes (STING) (14–17). The carboxyl terminus of stimulated STING recruits and activates TANK-binding kinase 1 (TBK1), which in turn phosphorylates the transcription factor interferon regulatory factor 3 (IRF3) (18, 19). The phosphorylated IRF3 dimerizes and then enters the nucleus to induce expression of IFNβ. Stimulated STING also activates I kappa B kinase, which phosphorylates the I kappa B (IkB) family of inhibitors of the nuclear factor-κB (NF-κB). NF-κB has the potential to cooperate with IRF3, thereby enhancing the maximal expression of IFNβ (20, 21).

The IFNβ response to herpesvirus infection is multifaceted, involving distinct temporal activation patterns at various stages of the viral life cycle, and plays important roles in herpesvirus infection (22–26). Therefore, herpesviruses have evolved to suppress the IFNβ pathway. Consistently, several studies have reported the impact of HHV-6 on IFNβ production. HHV-6 encodes immediate-early 1 (IE1) proteins that can suppress IFNβ production by disrupting key signaling molecules such as IRF3, TBK1, and mitochondrial antiviral-signaling protein (MAVS), thereby impairing antiviral gene expression (27, 28). HHV-6B IE1 further inhibits type I IFN responses by binding to signal transducer and activator of transcription 2 (STAT2), leading to nuclear accumulation of STAT2 and blockade of ISG expression (22). Additionally, HHV-6 infection alters cytokine profiles in infected monocytes and macrophages, increasing interleukin (IL)-10 while suppressing IL-12, thus shifting immune responses toward an anti-inflammatory state (29). The HHV-6B tegument protein U54 binds to the calcineurin phosphatase enzyme, disrupting the proper dephosphorylation and nuclear translocation of nuclear factor of activated T cells proteins, and thereby resulting in suboptimal IL-2 expression (30). However, the molecular mechanisms underlying the immune evasion strategies of HHV-6 remain incompletely understood.

In this study, we focused on the HHV-6B tegument protein U65, which is a homolog of HCMV UL94 (31, 32). We demonstrated that U65 serves as an inhibitor of the innate immune response related to particular host histone proteins, i.e., hCG_2039566 (herein referred to as H2ACG) and H2AC7. Overexpression of U65 suppressed the induction of IFNβ triggered by a viral DNA analog. U65 interacted with H2ACG and H2AC7, suppressing their ability to modulate the intensity of the antiviral response. Consequently, U65 impairs the H2ACG- and H2AC7-related antiviral pathways, thereby promoting HHV-6B replication. These findings enhance our understanding of the mechanisms of HHV-6B immune evasion during the infection.

## RESULTS

### HHV-6B U65 inhibits IFNβ production

**Fig 1:**
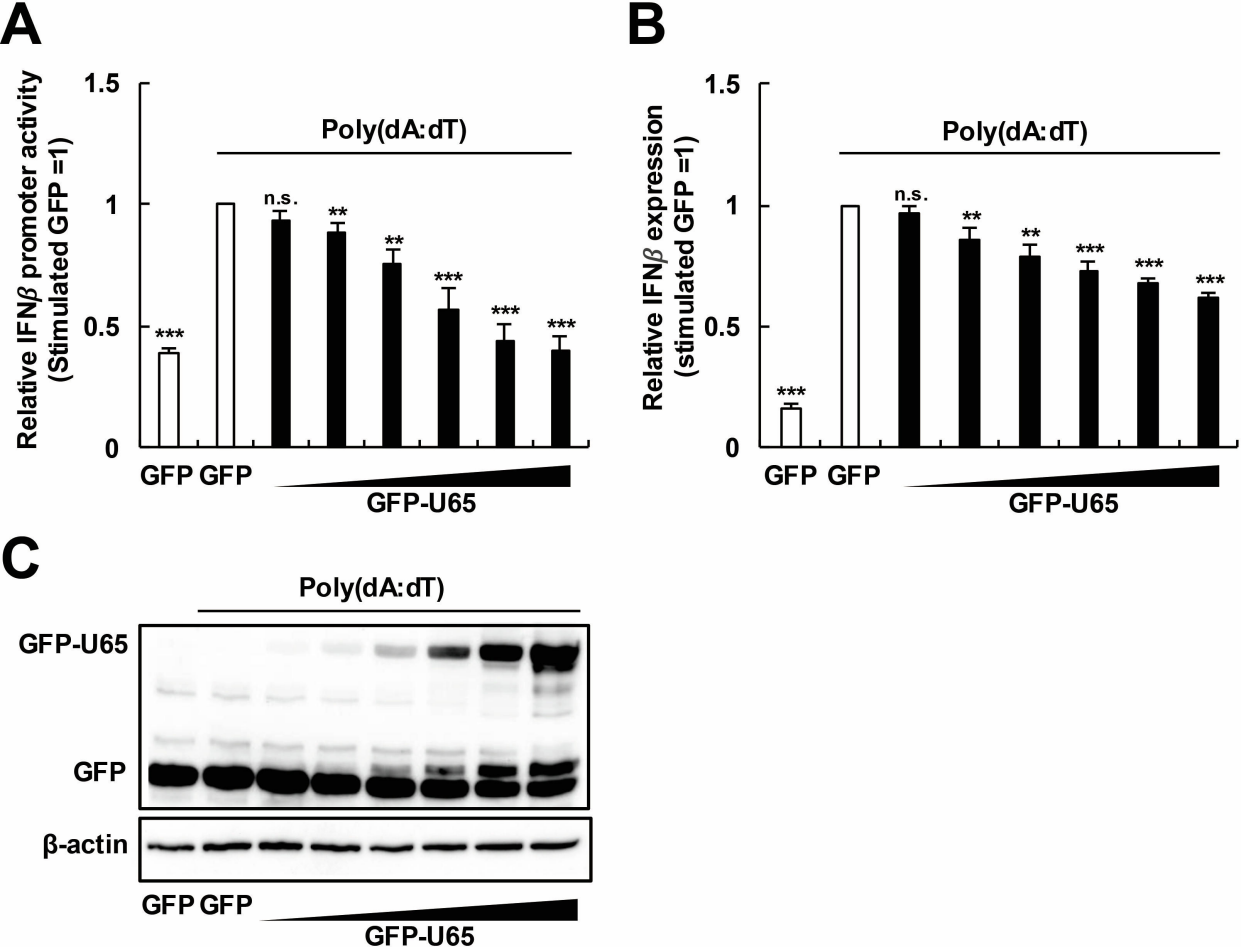
HHV-6B U65 inhibits IFNβ production. (**A**) Dose-dependent effect of U65 on the IFNβ promoter activity. 293T cells were transfected with an IFNβ promoter reporter plasmid (p125-luc) and pRL-TK, along with a GFP-expressing plasmid or increasing amounts (12.5–400 ng) of a GFP-U65-expressing plasmid. At 24 h post-transfection, the cells were stimulated with poly(dA:dT). Luciferase activity was measured as the IFNβ promoter activity. (**B**) Dose-dependent effect of U65 on the IFNβ expression level. Total RNA was extracted from the cells and subjected to reverse transcription quantitative PCR (RT-qPCR) analysis. (**C**) Dose-dependent expression of GFP-U65. The cell lysates were subjected to Western blot analysis with an anti-GFP antibody. A blot with an anti-β-actin antibody was included as a loading control. Values are expressed as the means + SE of at least four independent experiments. **, *P* < 0.01; ***, *P* < 0.001. n.s., no significance.

HHV-6B, a dsDNA virus, has evolved multiple strategies to antagonize innate immune pathways, thereby facilitating its replication (33–36). HHV-6B and HCMV belong to the same β-herpesvirus subfamily, and the HCMV tegument proteins, such as UL48, UL82/83, UL94, and UL97, are known to play roles in immune suppression (37–40). Among the homologous tegument proteins, the function of U65, a homolog of HCMV UL94, in HHV-6B remains unclear. Therefore, we hypothesized that U65 might promote viral infection by overcoming the restriction by innate immune responses. To investigate the role of U65 in the IFNβ pathway, we first explored whether U65 could suppress the activation of the IFNβ promoter by poly(deoxyadenylic-deoxythymidylic) acid [poly(dA:dT)], a synthetic viral dsDNA analog. 293T cells were transfected with an IFNβ promoter reporter plasmid (p125-luc), along with a green fluorescent protein (GFP)-expressing plasmid or increasing amounts of a GFP-U65-expressing plasmid. At 24 h post-transfection, the cells were stimulated with poly(dA:dT). Poly(dA:dT) activated the IFNβ promoter in GFP-expressing cells, whereas the activation of the IFNβ promoter was suppressed in GFP-U65-expressing cells in a dose-dependent manner (Fig. 1A). Since p125-luc is of murine origin, we then validated these results by measuring the human IFNβ mRNA levels. The expression of the IFNβ mRNA was robustly induced by poly(dA:dT) in GFP-expressing cells, whereas the induction was suppressed in GFP-U65-expressing cells in a dose-dependent manner (Fig. 1B). Dose-dependent expression of GFP-U65 was confirmed by Western blot analysis (Fig. 1C). These results suggest that HHV-6B U65 inhibits the poly(dA:dT)-triggered IFNβ production pathways.

### HHV-6B U65 does not affect the cytoplasmic IFNβ production pathway

**Fig 2:**
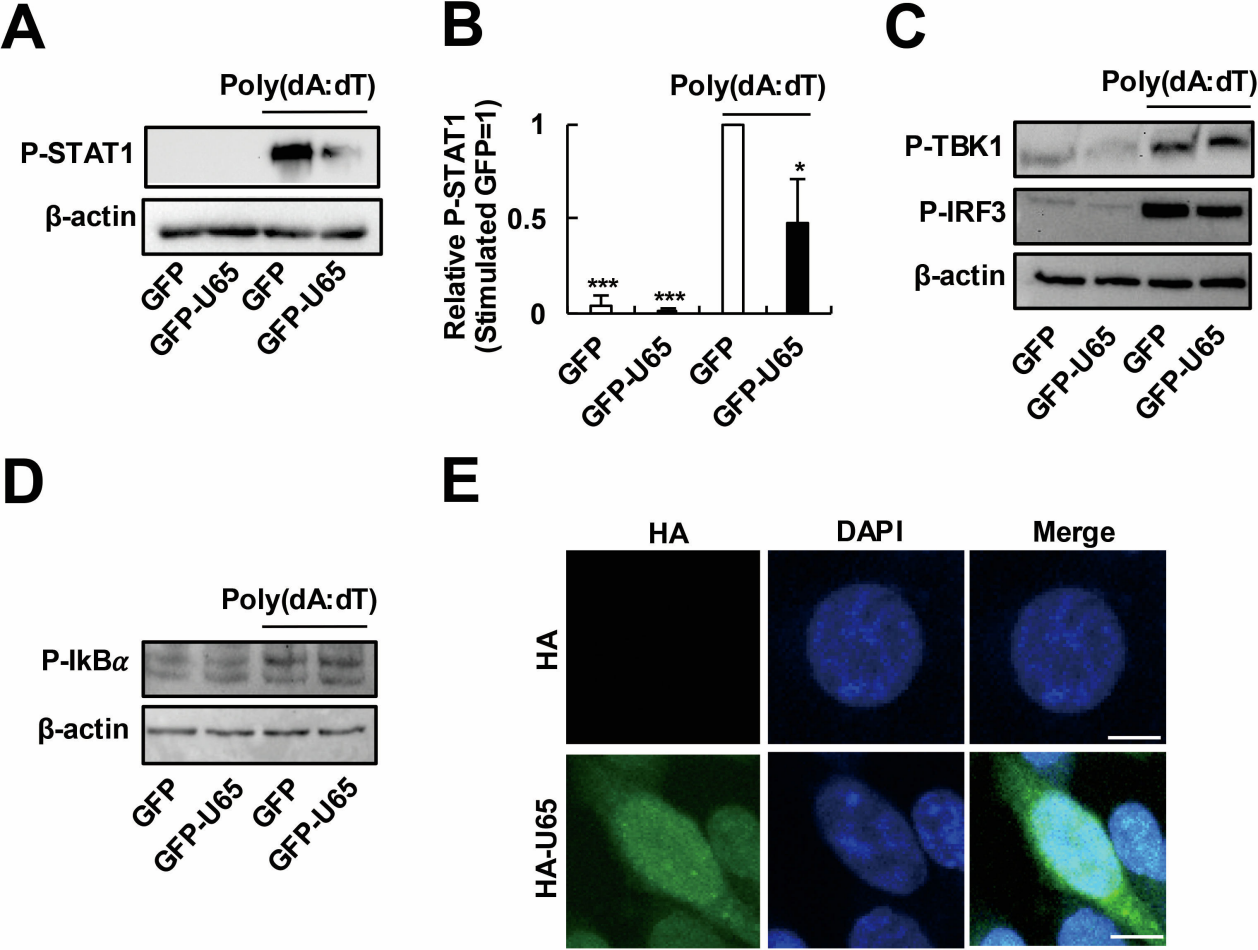
HHV-6B U65 does not affect the cytoplasmic IFNβ signaling pathway. (**A–D**) Effects of U65 on phosphorylation of STAT1 (A and B), TBK1 (**C**), IRF3 (**C**), and IkBα (D). 293T cells were transfected with a GFP-expressing plasmid or a GFP-U65-expressing plasmid. At 24 h post-transfection, the cells were stimulated with poly(dA:dT). The cell lysates, collected at 24 h (**A–C**) or 2 h post-stimulation (**D**), were subjected to Western blot analysis for phosphorylated STAT1 (A), TBK1 (**C**), IRF3 (**C**), and IkBα (**D**). A blot with an anti-β-actin antibody was included as a loading control. (**B**) Band intensities of phosphorylated STAT1 were quantified and normalized to β-actin levels. Values are expressed as the means + SE of at least three independent experiments. *, *P* < 0.05; ***, *P* < 0.001. (**E**) Localization of U65 in 293T cells. 293T cells were transfected with an HA-expressing plasmid or an HA-U65-expressing plasmid. At 48 h post-transfection, the cells were fixed and analyzed by immunofluorescence assay. Scale bars, 10 µm. DAPI, 4′,6-diamidino-2-phenylindole.

Given that HHV-6B U65 suppresses the IFNβ production pathway (Fig. 1), we investigated its effect on the phosphorylation of signal transducer and activator of transcription 1 (STAT1), a key downstream effector of the IFNβ signaling pathway (41). Consistent with the inhibitory effect of U65 on the IFNβ production pathway, U65 expression reduced STAT1 phosphorylation (Fig. 2A and B), indicating that U65 functionally suppresses the IFNβ signaling pathway. The cytosolic DNA sensing pathways, such as the cGAS–STING pathway, play a central role in the innate immune defense against herpesvirus infections (42). Therefore, we hypothesized that U65 might suppress innate immune response by inhibiting the DNA sensing pathways. To assess the effect of U65 on the DNA sensing pathways, we examined phosphorylation of TBK1, IRF3, and IkBα, which are hallmarks of activation of the cytoplasmic process in these pathways (18). U65 expression did not inhibit the poly(dA:dT)-triggered phosphorylation of TBK1, IRF3, and IkBα (Fig. 2C and D). These results suggest that U65 does not suppress the cytoplasmic process in the DNA sensing pathway. We then examined the intracellular localization of U65 and found that U65 localized both in the cytoplasm and the nucleus of transfected 293T cells (Fig. 2E). Taken together, these results suggest that nuclear-localizing U65 likely suppresses the nuclear process of the IFNβ production pathway.

### HHV-6B U65 suppresses the H2ACG- and H2AC7-related IFNβ pathways

**Fig 3:**
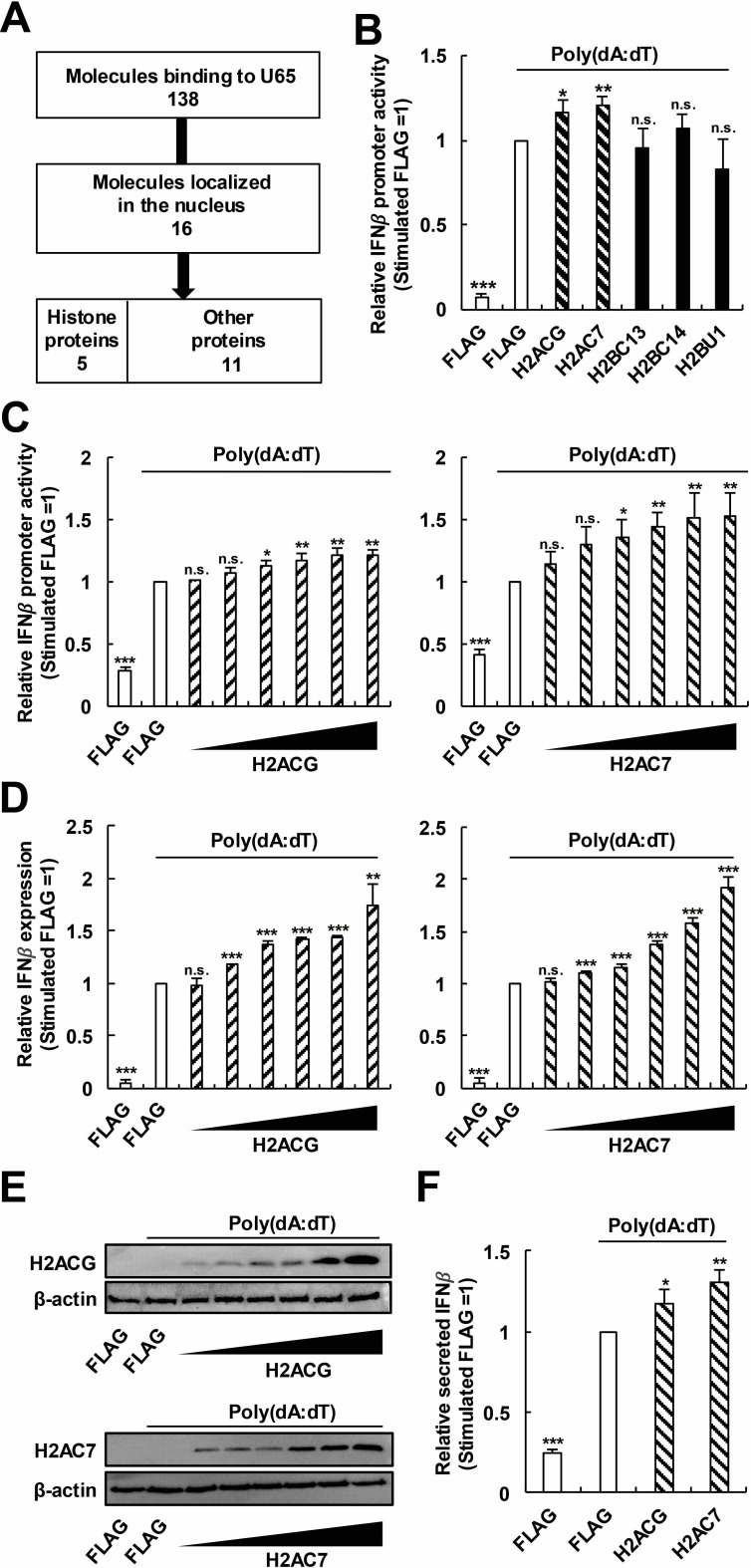
H2ACG and H2AC7 enhance the poly(dA:dT)-triggered IFNβ pathway. (**A**) Flow diagram of candidate molecule selection. A total of 138 candidate molecules were identified using liquid chromatography–mass spectrometry as U65-binding proteins. Of these, 16 molecules were localized in the nucleus. Among them, we noticed that candidate molecules contained five histone proteins. Because of this enrichment and the fact that particular histones are reported to induce IFNβ production, we selected five histone proteins for further investigation. (**B**) Effects of histone proteins on the IFNβ promoter activity. 293T cells were transfected with p125-luc and pRL-TK, along with each histone-expressing plasmid (FLAG-H2ACG, H2AC7, H2BC13, H2BC14, or H2BU1). At 24 h post-transfection, the cells were stimulated with poly(dA:dT). (**C**) Dose-dependent effects of histone proteins on the IFNβ promoter activity. 293T cells were transfected with increasing amounts (50–1,600 ng) of a histone-expressing plasmid (FLAG-H2ACG or H2AC7), along with p125-luc and pRL-TK. The cells were stimulated with poly(dA:dT) for 18 h. (**D**) Dose-dependent effects of histone proteins on the IFNβ expression level. 293T cells were transfected with increasing amounts (50–1,600 ng) of each histone-expressing plasmid (FLAG-H2ACG or H2AC7). The cells were stimulated with poly(dA:dT) for 18 h. Total RNA was extracted for RT-qPCR analysis. (**E**) Dose-dependent expression of FLAG-H2ACG or H2AC7. The cell lysates were subjected to Western blot analysis with an anti-FLAG antibody. A blot with an anti-β-actin antibody was included as a loading control. (**F**) Effects of histone proteins on the IFNβ protein level. 293T cells were transfected with each histone-expressing plasmid (FLAG-H2ACG or H2AC7). The cells were stimulated with poly(dA:dT) for 18 h. Culture media were collected for enzyme-linked immunosorbent assay of secreted IFNβ. Values are expressed as the means + SE of at least four independent experiments. *, *P* < 0.05; **, *P* < 0.01; ***, *P* < 0.001. n.s., no significance.

We next investigated the molecular mechanisms by which U65 suppresses the nuclear IFNβ pathway by identifying U65-binding proteins. We expressed GFP or GFP-U65 in 293T cells, immunoprecipitated using an anti-GFP nanobody, determined coimmunoprecipitated proteins by liquid chromatography–mass spectrometry (LC-MS), and then identified proteins coprecipitated with GFP-U65 but not with GFP. Since U65 likely inhibits the nuclear IFNβ production pathway (Fig. 2), we selected 16 nuclear-localizing proteins among the identified U65-binding proteins. Notably, they included five histone variant proteins (Fig. 3A and Table 1). Histones act in the nucleus and particular histone variant proteins are reported to regulate IFNβ production (43–48). Based on these reports, the enrichment of histone proteins in our candidates prompted us to further investigate the involvement of these histones in the IFNβ production pathway.

**TABLE 1:** The prot_score and the prot_matches in the samples precipitated with GFP or GFP-U65^*a*^

Accession number	Description	Prot_score (GFP)	Prot_matches (GFP)	Prot_score (GFP-U65)	Prot_matches (GFP-U65)
Q8N257	Histone H2B type 3-B (H2BU1)	45	1	591	35
Q99879	Histone H2B type 1-M (H2BC14)	347	8	620	42
Q99880	Histone H2B type 1-L (H2BC13)	347	8	596	41
A0A0U1RR32	Histone H2A (hCG_2039566)	154	4	336	24
P20671	Histone H2A type 1-D (H2AC7)	154	4	336	24

^*a*^ Prot_score is a protein score that was calculated using Mascot software. Prot_matches are the numbers of peptides that were identified in the samples.

We first examined whether identified histone proteins affect the IFNβ production pathway in our experimental setting. 293T cells were transfected with p125-luc, along with each histone protein-expressing plasmid (H2ACG, H2AC7, H2BC13, H2BC14, or H2BU1). In our experimental setting, expression of H2ACG or H2AC7 promoted the poly(dA:dT)-triggered activation of the IFNβ promoter (Fig. 3B). We further confirmed that H2ACG or H2AC7 expression promoted the IFNβ promoter activity and the IFNβ mRNA expression activated by poly(dA:dT) in a dose-dependent manner (Fig. 3C through E). The effect of H2ACG or H2AC7 expression on the IFNβ production was also confirmed at the protein level in culture media using enzyme-linked immunosorbent assay (ELISA) (Fig. 3F).

**Fig 4:**
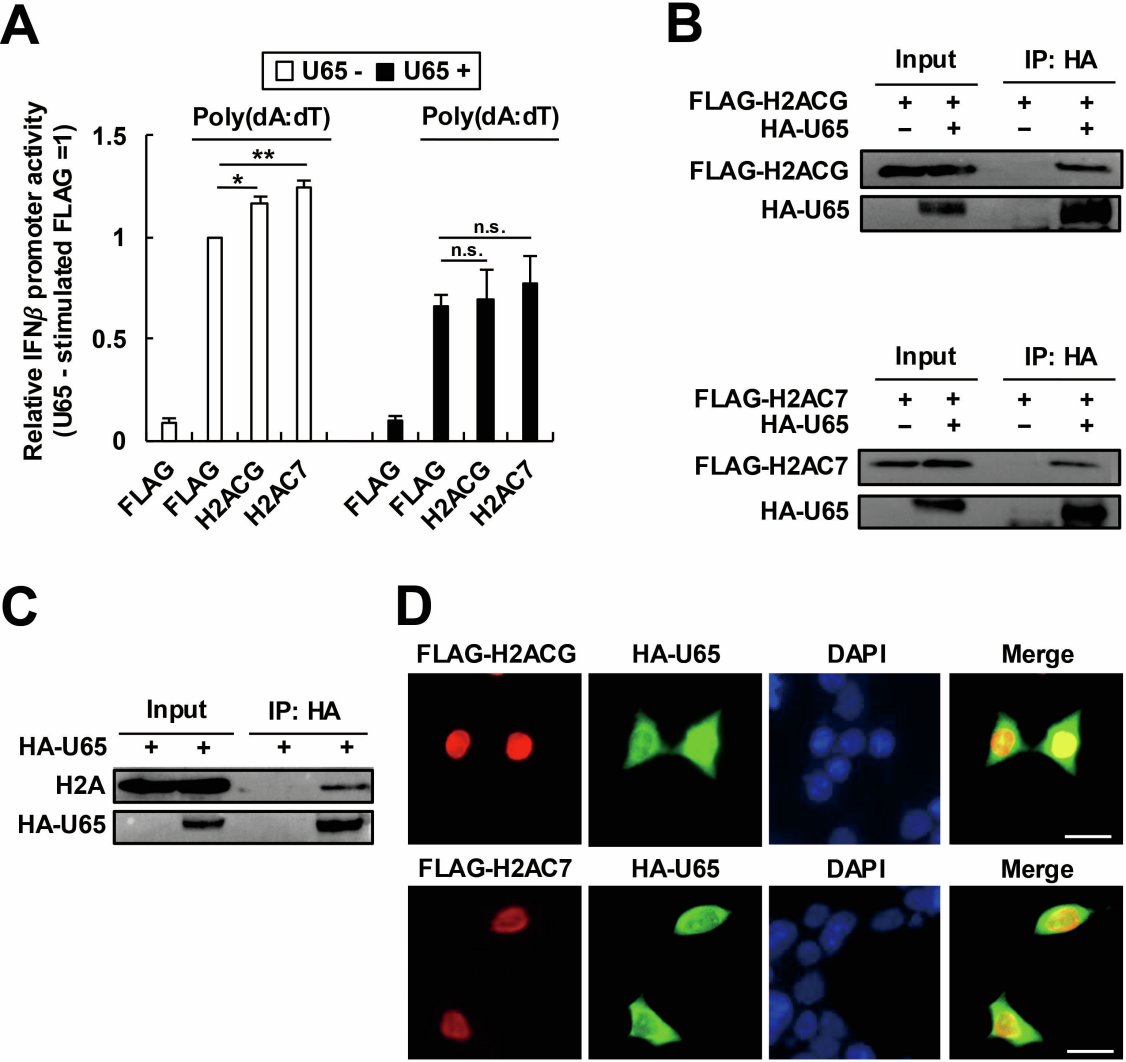
HHV-6B U65 suppresses the H2ACG- and H2AC7-related IFNβ pathway. (**A**) Effects of U65 on the IFNβ promoter activity related to H2ACG or H2AC7. 293T cells were transfected with p125-luc, pRL-TK, and each histone-expressing plasmid (FLAG-H2ACG or H2AC7), in the presence or absence of HA-U65. The cells were stimulated with poly(dA:dT). Luciferase activity was measured as the IFNβ promoter activity. Values are expressed as the means + SE of at least four independent experiments. *, *P* < 0.05; **, *P* < 0.01. n.s., no significance. (**B**) Immunoprecipitation of H2ACG or H2AC7 with U65. 293T cells were transfected with plasmids expressing HA-U65 and FLAG-H2ACG or H2AC7. The cell lysates were immunoprecipitated with an anti-FLAG antibody, and the precipitated proteins were blotted with an anti-HA or an anti-FLAG antibody. (**C**) Immunoprecipitation of endogenous H2A with U65. 293T cells were transfected with a HA-U65-expressing plasmid. The cell lysates were immunoprecipitated with an anti-HA antibody, and the precipitated proteins were blotted with an anti-HA or an anti-H2A antibody. (**D**) Codistribution of H2ACG or H2AC7 with U65. 293T cells were transfected with plasmids expressing HA-U65 and FLAG-H2ACG or H2AC7. The cells were fixed and stained with anti-HA and anti-FLAG antibodies. Scale bars, 20 µm.

We then examined the effect of U65 on the H2ACG- or H2AC7-mediated IFNβ promoter regulation. Expression of H2ACG or H2AC7 promoted the activation of IFNβ promoter in the absence of U65 (Fig. 4A, left). On the other hand, the expression of these histone proteins did not promote the activation of the IFNβ promoter in the presence of U65 (Fig. 4A, right). Furthermore, we confirmed the interaction of H2ACG or H2AC7 with U65 using coimmunoprecipitation (Fig. 4B). To obtain physiological relevance, we also demonstrated the interaction between endogenous H2A and U65 although the antibody for H2A used in this study is not specific to H2ACG and H2AC7 (Fig. 4C). Consistent with the results described above, the codistribution of H2ACG or H2AC7 with U65 was observed in an immunofluorescence assay (IFA) (Fig. 4D). Taken together, our results indicate that the HHV-6B tegument protein U65 inhibits innate immune responses by interfering with the IFNβ production influenced by H2ACG and H2AC7.

### HHV-6B U65 contains a peptide sequence with similarity to histone proteins

**Fig 5:**
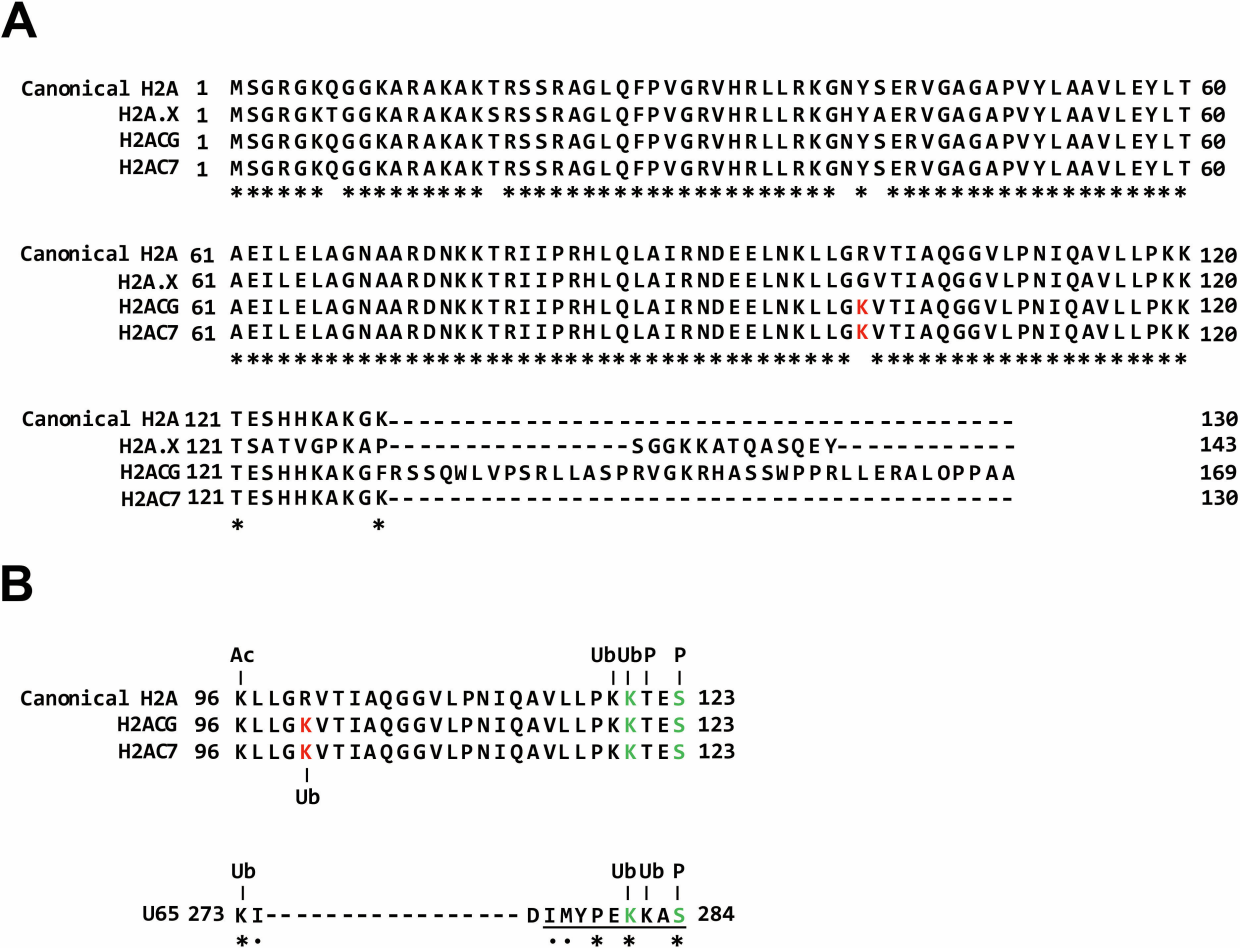
Sequence alignment and post-translational modification (PTM) site prediction of histone proteins and U65. (**A**) Sequence alignment of histone variants (H2A.X, H2ACG, and H2AC7) with canonical histone H2A. Amino acid sequences of canonical histone H2A and its variants (H2A.X, H2ACG, and H2AC7) were aligned using MAFFT. Conserved residues are marked with asterisks (*). Lysine (K) residues unique to H2ACG and H2AC7, compared to canonical H2A, are highlighted in red. (**B**) Prediction of PTM sites in canonical histone H2A, H2ACG, H2AC7, and U65. PTM sites, including phosphorylation, ubiquitination, acetylation, and methylation, were predicted using MusiDeep. The underlined peptide sequence (positions 276–284) in U65 is analogous to the C-terminal histone tail of H2A, H2ACG, and H2AC7 (positions 115–123). Conserved residues and residues with similar properties are indicated by asterisks (*) and dots (.), respectively. Predicted phosphorylation (P), ubiquitination (Ub), and acetylation (Ac) sites are annotated accordingly. K residues unique to H2ACG and H2AC7, compared to canonical H2A, are highlighted in red. K and serine (S) residues predicted to undergo ubiquitination and phosphorylation, respectively, in both histone proteins and U65 are highlighted in green.

To gain insights into the underlying mechanism, we compared the amino acid sequences of H2ACG and H2AC7 with those of canonical H2A and the well-known variant H2A.X (Fig. 5A). We identified that H2ACG and H2AC7 share a lysine (K) residue at position 100, which is substituted with arginine in canonical H2A and glycine in the H2A.X variant (Fig. 5A, highlighted in red). This suggests that the presence of additional lysine residues in H2ACG and H2AC7 may allow for post-transcriptional modifications (PTMs), potentially contributing to the induction of IFNβ expression (43, 49, 50). Consistently, the lysine residue unique to H2ACG and H2AC7 is predicted to be ubiquitinated (Fig. 5B). Next, to evaluate the possibility that U65 mimics histone proteins, we compared the amino acid sequence of U65 with canonical H2A, H2ACG, and H2AC7 (Fig. 5B). Sequence alignment revealed that U65 contains a peptide with similarity to the C-terminal histone tail domain of H2A proteins (Fig. 5B, underlined). Notably, a K residue at position 281 and a serine (S) residue at position 284 in U65 are predicted to undergo ubiquitination and phosphorylation, respectively, analogous to the ubiquitination and phosphorylation observed in canonical H2A (51, 52). These results support the hypothesis that U65 may function as a histone mimic in U65-mediated immune evasion.

### Knockdown of HHV-6B U65 restores IFNβ production and reduces viral load during HHV-6B infection

**Fig 6:**
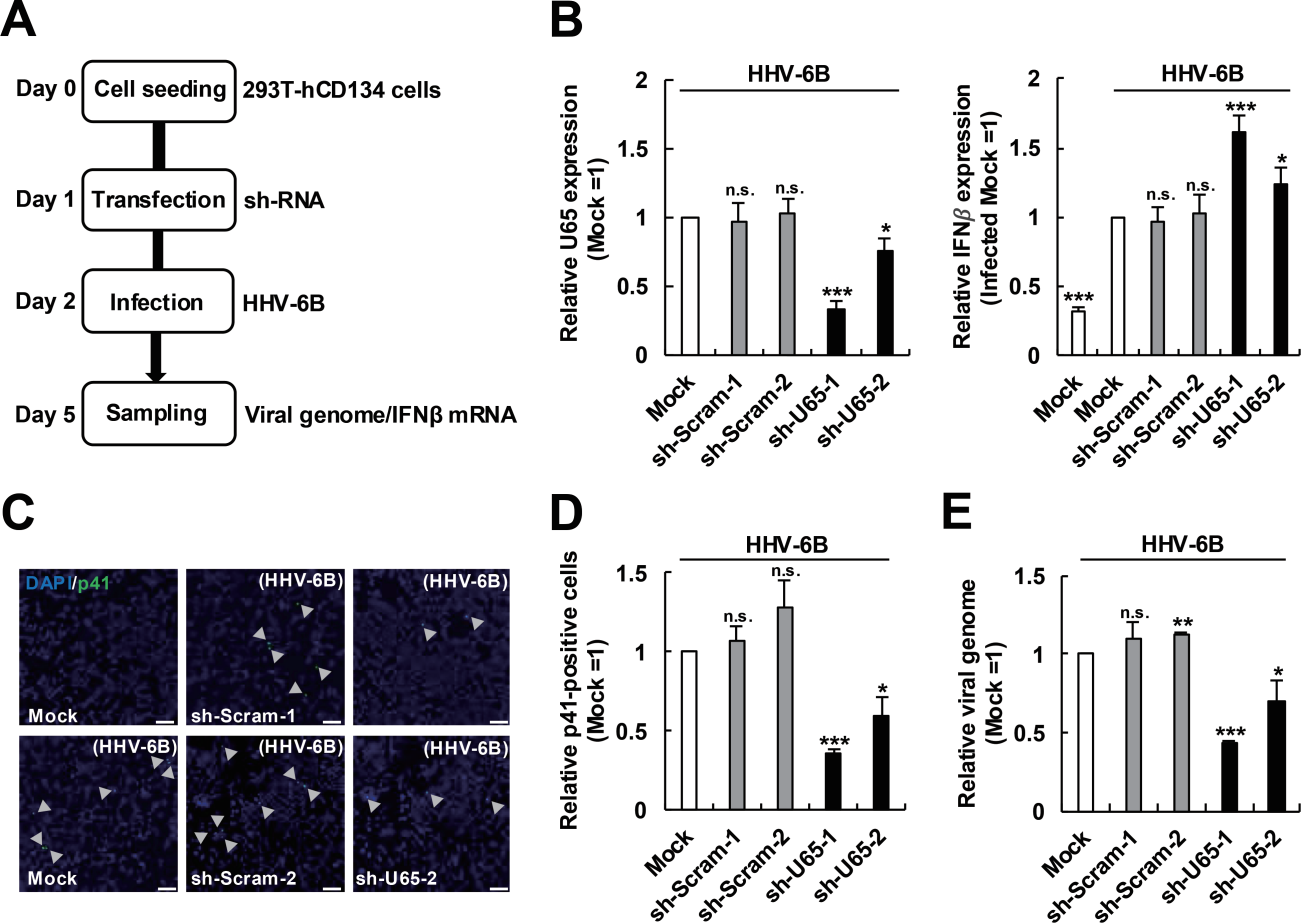
Knockdown of HHV-6B U65 restores IFNβ production and reduces viral load during HHV-6B infection. (**A**) Schematic view of the experimental procedure. 293T-hCD134 cells were transfected with a sh-Scram-expressing plasmid or a sh-U65-expressing plasmid. At 24 h post-transfection, the cells were infected with HHV-6B (MOI of 0.1) and further incubated for 3 days. (**B**) Effects of U65 knockdown on the expression levels of U65 and IFNβ. Total RNA of infected 293T-hCD134 cells was extracted and subjected to RT-qPCR analysis for the mRNA level of U65 and IFNβ. (**C**) IFA of infected 293T-hCD134 cells. 293T-hCD134 cells were stained with a mouse antibody against HHV-6 p41 early antigen (9A5D12) and DAPI. Green, HHV-6B p41; blue, DAPI. Arrowheads, p41-positive infected cells. Scale bars, 100 µm. (**D**) The number of infected 293T-hCD134 cells. (**E**) The viral load in infected 293T-hCD134 cells. Total DNA of infected 293T-hCD134 cells was extracted and subjected to quantitative PCR analysis for HHV-6B genome DNA. Values are expressed as the means + SE of at least three independent experiments. *, *P* < 0.05; **, *P* < 0.01; ***, *P* < 0.001; n.s., no significance.

We finally examined the role of U65 in the inhibition of IFNβ production during HHV-6B infection. 293T-hCD134 cells, a cell line stably expressing an HHV-6 entry receptor, human CD134, were first transfected with a sh-U65-expressing plasmid and then infected with the HHV-6B Z29 strain at a multiplicity of infection (MOI) of 0.1 (Fig. 6A). Knockdown of U65 by sh-U65-1 or sh-U65-2 successfully decreased U65 mRNA expression (Fig. 6B, left). In this setting, IFNβ mRNA expression following HHV-6B Z29 infection was increased by U65 knockdown (Fig. 6B, right), consistent with the results described above, and the number of the infected cells (p41-positive cells) or the viral load was reduced as expected (Fig. 6C through E). Altogether, these results demonstrate that U65 mediates immune evasion during HHV-6B infection to support efficient infection.

## DISCUSSION

Herpesviruses constitute a large family of enveloped dsDNA viruses that establish a lifelong persistent infection in the host. The viral DNA in the infected cells is recognized by PRRs, which trigger IFNβ production and subsequent antiviral responses. These responses inhibit viral replication and promote the elimination of infected cells (15, 17, 18, 53). On the other hand, herpesviruses have developed diverse strategies to counteract the innate immune responses. For example, Kaposi’s sarcoma-associated herpesvirus (KSHV) tegument protein ORF52 has been shown to inhibit the enzymatic activity of cGAS, disrupting the synthesis of cGAMP (54). Similarly, the KSHV tegument protein ORF33 interacts with STING and MAVS and facilitates the recruitment of host protein phosphatase, Mg^2+^/Mn^2+^-dependent 1G to dephosphorylate phosphorylated STING and MAVS, thereby suppressing immune responses (55). The Epstein–Barr virus (EBV) tegument protein BGLF2 has been identified as a potent suppressor of the Janus kinase-signal transducer and activator of transcription pathway, through which ISGs are induced (56). Additionally, the HCMV tegument protein UL94 has been shown to interact with mediator of IRF3 activation (MITA), impairing the recruitment of TBK1 to the MITA microsome and suppressing the induction of IFNβ triggered by cytosolic dsDNA and DNA viruses (38).

Based on the comparison with the homologous HCMV tegument proteins known for their inhibitory effects on the antiviral innate immune response, we selected to investigate HHV-6B U65 in this study as the effect of U65 on IFNβ production has not been reported. The HHV-6B tegument protein U65 is homologous to HCMV UL94 that acts as a core herpesvirus structural component to facilitate the secondary envelopment of virions and targets MITA to disrupt the recruitment of TBK1 to the MITA microsome, thereby evading the antiviral immune response (38, 57). Expression of U65 suppressed the poly(dA:dT)-triggered IFNβ induction (Fig. 1A and B). Conversely, knockdown of U65 enhanced HHV-6B-induced IFNβ production (Fig. 6B). Additionally, we demonstrated that knockdown of U65 reduced viral replication during HHV-6B infection (Fig. 6C through E). Collectively, HHV-6B U65 plays a critical role in immune evasion during HHV-6B infection.

**Fig 7:**
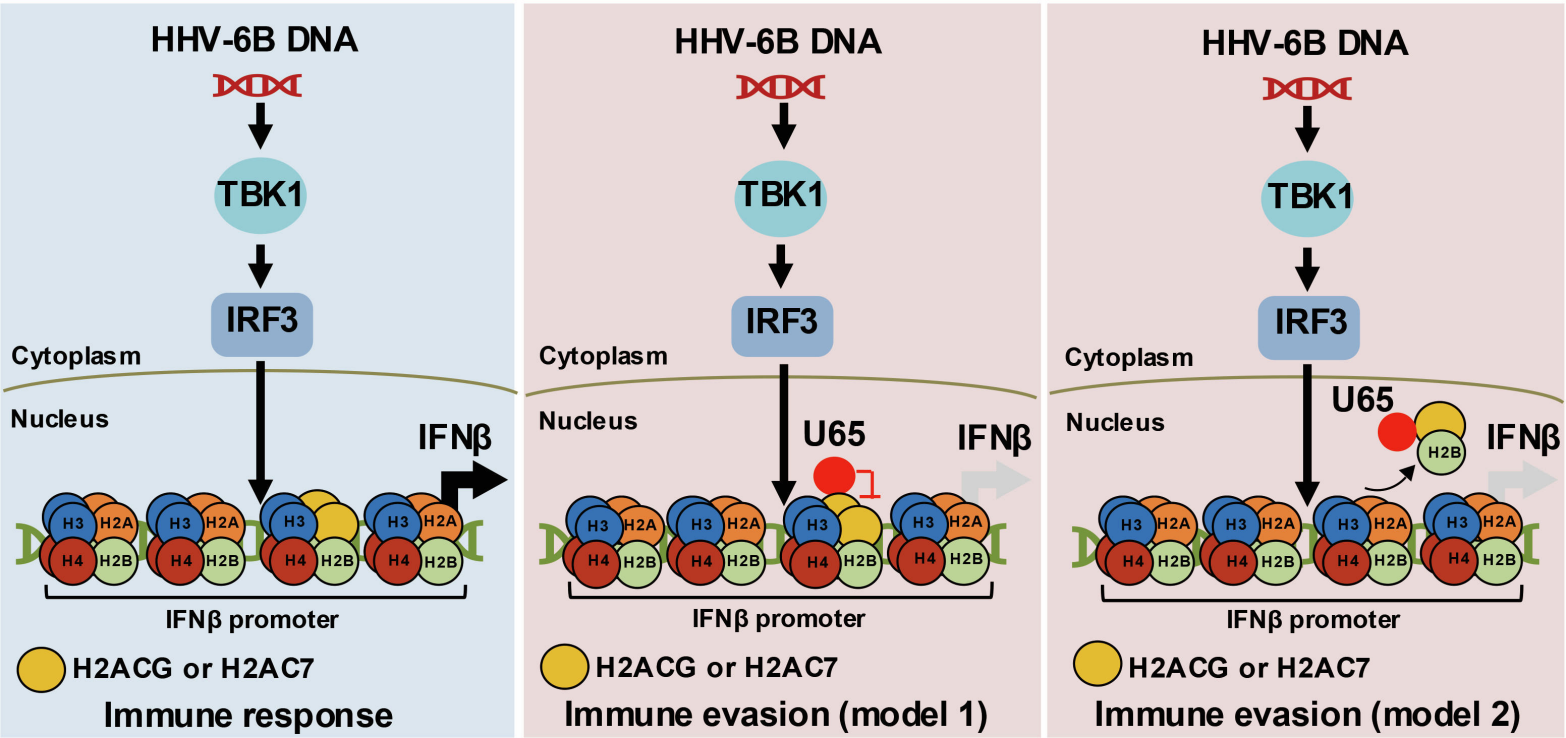
Models for U65-mediated immune evasion. In the presence of HHV-6B DNA, TBK1 is activated to phosphorylate IRF3, causing it to dimerize and move into the nucleus, where it stimulates transcription of the *IFNβ* gene. The histone proteins, H2ACG and H2AC7, promote the IFNβ production pathway in the nucleus (left). However, this system is hijacked by HHV-6B for immune evasion. During HHV-6B infection, the tegument protein U65 interacts with H2ACG and H2AC7, impairing the function of H2ACG and H2AC7 to promote the IFNβ pathway (middle). Alternatively, U65 suppresses H2ACG and H2AC7 incorporation into the IFNβ promoter, thereby impairing the H2ACG- and H2AC7-mediated regulation of the IFNβ production (right).

Histones are essential nuclear proteins that form the nucleosome, the fundamental structural unit of chromatin fibers in eukaryotes (58), and play key roles in regulating gene expression (59). The nucleosome, composed of an octamer of core histones—two each of H2A, H2B, H3, and H4—serves as the basic unit of chromatin organization (60). Structurally, the histone octamer is organized such that an H3–H4 tetramer forms the central scaffold, flanked by two H2A–H2B dimers. Each H2A–H2B dimer interacts with the H3–H4 tetramer to create a “dimer of dimers” arrangement, stabilizing the DNA–protein complex (61, 62). This architecture not only provides a compact packaging system for DNA but also serves as a dynamic platform for epigenetic regulation through histone modifications and variant incorporation (63, 64). Multiple nucleosomes form higher-order chromatin fibers whose compaction states influence transcriptional activity (65). Thus, while the nucleosome represents the primary level of chromatin organization, the collective arrangement of nucleosomes and their associated proteins defines the structural and functional landscape of chromatin in the nucleus (66). Within the nucleosomes, histone variants such as H2A.X, H2A.Z, and macroH2A can replace canonical H2A, modulating chromatin structure and function (67). Recent studies have shown that nucleosomes containing the histone variant H2A.Z recruit BRD2, which facilitates transcriptional repression and suppresses IFNβ responses, potentially contributing to immune evasion (43, 68). In this study, we noticed that 5 of 16 identified U65-binding nuclear proteins were histone variant proteins (Fig. 3A and Table 1) (60). We found that overexpression of histone proteins, H2ACG or H2AC7, promoted the production of IFNβ (Fig. 3B through F). To explore the potential mechanisms, we performed amino acid sequence alignment of H2ACG and H2AC7 with canonical H2A and the well-characterized variant H2A.X (Fig. 5A). We identified a K residue at position 100 unique to H2ACG and H2AC7. Given that lysine residues are often subjected to PTMs in histone proteins (69), we hypothesize that this lysine may undergo modifications, e.g., ubiquitination (predicted in Fig. 5B), potentially promoting IFNβ production (Fig. 7, left). Further investigation is needed to test this hypothesis.

U65 compromises the innate immune response to HHV-6B by targeting the H2ACG- and H2AC7-mediated regulation of the IFNβ pathway at least in part (Fig. 4). U65 may inhibit the function of histone variants, H2ACG and H2AC7, through two distinct mechanisms (Fig. 7). First, U65 may function as a mimic of H2ACG and H2AC7, disrupting their regulation of the IFNβ pathway (Fig. 7, middle). U65 contains a peptide sequence (positions 276–284) analogous to the histone tail of these H2A variants (positions 115–123) (Fig. 5B), which may act as a histone mimic by potentially undergoing competitive ubiquitination at the K residue at position 281 and phosphorylation at the S residue at position 284 in U65, thereby suppressing ubiquitination of the K residue at position 121 and phosphorylation of the S residue at position 123 in the H2ACG and H2AC7, respectively. In this context, in addition to the K residue at position 100, ubiquitination of the K residue at position 121 and phosphorylation of the S residue at position 123 in H2ACG and H2AC7 are likely required for their function. Alternatively, U65 may inhibit the incorporation of the IFNβ-enhancing histone variants, H2ACG and H2AC7, into the IFNβ promoter, thereby suppressing IFNβ production (Fig. 7, right). These models are plausible because recent studies have demonstrated that histone modulation by viral proteins, such as mimicking or hijacking host histone regulatory machineries, is a common strategy for efficient viral infection (70). For example, the influenza virus encodes a histone mimic that suppresses antiviral responses by disrupting host transcriptional programs (71, 72). The HSV protein ICP0 facilitates histone removal from viral DNA during lytic infection (73), while it also induces chromatin de-repression of latent viral genomes through modulation of host histone marks (74). In EBV, the histone variant H2A.Z cooperates with the EBV latency maintenance protein EBNA1 to sustain a viral chromatin landscape during latency (75).

In summary, although the mechanism on how U65 compromises the H2ACG- and H2AC7-mediated regulation of IFNβ production remains unknown, we identified previously uncharacterized roles of U65 as an inhibitor of these histones in the immune evasion of HHV-6B during the infection. These findings expand our understanding of the mechanisms employed by HHV-6B to ensure persistent infection and enhance our knowledge of the virus–host interplay. A limitation of this study is that HHV-6B infection was evaluated only in 293T-hCD134 cells. Future validation using cell lines commonly employed for HHV-6B infection, such as MT-4 cells, is needed. Given the role of U65 in immune evasion and potential involvement in secondary virion envelopment, therapeutic strategies targeting U65 represent a promising approach to controlling HHV-6B infection.

## MATERIALS AND METHODS

### Cells

293T cells (a human embryonic kidney cell line, ATCC #CRL-3216) and 293T-hCD134 cells, which are overexpressing human CD134 to facilitate HHV-6B infection (76–78), were cultured in Dulbecco’s modified Eagle’s medium (DMEM) supplemented with 5% fetal bovine serum at 37°C and 5% CO_2_.

### Antibodies and compounds

Rabbit antibodies against human phosphorylated TBK1 Ser172 (clone: D52C2), phosphorylated IRF3 Ser386 (clone: E7J8G), phosphorylated IkBα Ser32 (clone: 14D4), phosphorylated STAT1 Tyr701 (clone: 9167), and histone H2A (clone: 2578) were purchased from Cell Signaling Technology (Danvers, MA, USA). Mouse antibodies against β-actin (clone: AC-15) and anti-HHV-6 p41 early antigen (clone: 9A5D12) were purchased from FUJIFILM Wako (Osaka, Japan) and Santa Cruz Biotechnology (Dallas, TX, USA), respectively. A rabbit antibody against FLAG (F7425) was purchased from Sigma-Aldrich (Merck KGaA, Darmstadt, Germany). Mouse antibodies against hemagglutinin (HA) (M180-3) and FLAG (M185-3L) were purchased from MBL (Nagoya, Japan). Alexa Fluor 488-conjugated goat anti-mouse IgG (H + L) F(ab′)_2_ (A-11017) and Alexa Fluor 555-conjugated goat anti-mouse IgG (H + L) F(ab′)_2_ (A-21425) were purchased from Thermo Fisher Scientific (Waltham, MA, USA). Horseradish peroxidase (HRP)-conjugated goat anti-mouse IgG (H + L) (115-035-062) was purchased from Jackson ImmunoResearch (West Grove, PA, USA). HRP-conjugated anti-rabbit IgG (H + L) (catalog no. 474-1516) was purchased from KPL (SeraCare Life Sciences, Milford, MA, USA). Anti-HA-tag mAb-Magnetic Agarose (M132-10), poly(dA:dT) (catalog no. tlrl-pic, tlrl-patn-1), and polyethylenimine “Max” were purchased from MBL, InvivoGen (Toulouse, France), and Polysciences (Warrington, PA, USA), respectively.

### Plasmids

Human cDNAs encoding H2ACG, H2AC7, H2BC13, H2BC14, H2BU1, and the HHV-6B tegument U65 were cloned into three different vectors (pCMV-GFP, pCAGSS-HA, and pCAGSS-FLAG) based on the required tags. For a plasmid expressing shRNA against U65 (sh-U65) or that encoding scramble sequences (sh-Scram), pairs of oligos (sh-U65-1, 5′-ACC GGG GAA TAA TGA AAT TTA AAT ACC TGA CCC ATA TTT AAA TTT CAT TAT TCC CTT TTT-3′ and 5′-CGA AAA AAA GGG AAT AAT GAA ATT TAA ATA TGG GTC AGG TAT TTA AAT TTC ATT ATT CCC-3′; sh-U65-2, 5′-ACC GGA AGA ATA AGC CTA AGA AAT AGC TTC CTG TCA CTA TTT CTT AGG CTT ATT CTT CTT TT-3′ and 5′-CGA AAA AAG AAG AAT AAG CCT AAG AAA TAG TGA CAG GAA GCT ATT TCT TAG GCT TAT TCT TC-3′; sh-Scram-1, 5′-ACC GGA AAG ATT AAG ATT GAT AAT ACC TGA CCC ATA TTA TCA ATC TTA ATC TTT CTT TTT-3′ and 5′-CGA AAA AAA GAA AGA TTA AGA TTG ATA ATA TGG GTC AGG TAT TAT CAA TCT TAA TCT TTC-3′; and sh-Scram-2, 5′-ACC GGA AGA AAT CAT AGA GAC TAA AGC TTC CTG TCA CTT TAG TCT CTA TGA TTT CTT CTT TT-3′ and 5′-CGA AAA AAG AAG AAA TCA TAG AGA CTA AAG TGA CAG GAA GCT TTA GTC TCT ATG ATT TCT TC-3′) were annealed and inserted into the BbsI sites of pRSI9-U6-(sh)-UbiC-RFP-2A-Puro (Cellecta). The reporter plasmid for the IFNβ promoter assay, p125-luc, was kindly provided by Prof. Fujita (Kyoto University) (79).

### Luciferase reporter assays

293T cells were transfected with p125-Luc and pRL-TK (Promega, Fitchburg, WI, USA), along with the indicated expression plasmids, and incubated for 24 h at 37°C and 5% CO_2_. At 24 h post-transfection, the cells were further transfected with 0.4 µg of poly(dA:dT) using polyethylenimine "Max" (1 mg/mL) and lysed using passive lysis buffer (TOYO INK, Tokyo, Japan) at 18 h post-poly(dA:dT) stimulation. Luciferase assays were performed as previously described (80) with some modifications using the PicaGene Dual Sea Pansy Luminescence Kit (Wako, catalog no. 301-05584), in which luciferase activity was quantified with the LUMAT3 LB 9508 luminometer (Berthold, Bad Wildbad, Germany). Relative luciferase activity was calculated by normalizing *Firefly* luciferase activity to *Renilla* luciferase activity.

### Real-time PCR (quantitative PCR) analyses

Reverse transcription real-time quantitative PCR (RT-qPCR) was performed as previously described (81–83) with minor modifications. Total RNA from 293T cells was extracted using TRI Reagent (Molecular Research Center, Cincinnati, Ohio, USA) according to the manufacturer’s protocol. mRNA was reverse transcribed into cDNA using a Verso cDNA Synthesis Kit (Thermo Fisher Scientific) with oligo-dT primers. The relative mRNA levels of human IFNβ were measured using a CFX Connect Real-Time System (Bio-Rad Laboratories, USA) with THUNDERBIRD Next SYBR (TOYOBO, Osaka, Japan) and normalized to GAPDH (glyceraldehyde-3-phosphate dehydrogenase) expression. Total DNA was extracted from infected cells using QIAamp DNA Mini Kit (Qiagen, Hilden, Germany) according to the manufacturer’s protocol. Quantitative PCR (qPCR) assays for HHV-6B genomic DNA were carried out using THUNDERBIRD Next SYBR (TOYOBO) and the HHV-6B-specific primers. The gene-specific primers used in this study are as follows: human IFNβ forward, 5′-GCC GCA GTG ACC ATC TAT GA-3′; human IFNβ reverse, 5′-CTC ATG CGT TTT CCC CTG GT-3′; human GAPDH forward, 5′-AGC GAG ATC CCT CCA AAA TC-3′ (84); human GAPDH reverse, 5′-AAA TGA GCC CCA GCC TTC TC-3′ (84); HHV-6B-U65 forward, 5′-TTG CAT GCA TTG CGA GAT GG-3′; HHV-6B-U65 reverse, 5′-GCT CCG GTG TAA CAC AAT GC-3′; HHV-6B-genome forward, 5′-TTT GCA GTC ATC ACG ATC GG-3′ (85); HHV-6B genome reverse, 5′-AGA GCG ACA AAT TGG AGG TTT C-3′ (85).

### Western blot

Western blot was performed as previously described (86) with some modifications. Briefly, 293T cells were lysed in sodium dodecyl sulfate (SDS) sample buffer. The cell lysates were separated by sodium dodecyl sulfate–polyacrylamide gel electrophoresis (SDS-PAGE) and transferred onto polyvinylidene difluoride membranes (Millipore, Bedford, MA, USA). The membranes were then blocked with 3% skim milk in phosphate-buffered saline (PBS) containing 0.05% Tween 20 for 1 h at room temperature, followed by incubation with primary antibodies for 1 h at room temperature. After washing, the membranes were incubated with secondary antibodies. The bound antibodies were visualized using Clarity Western ECL Substrate (Bio-Rad) and detected with a MultiImager II (BioTools, Gunma, Japan).

### Identification of U65-binding proteins

GFP-tagged U65- or GFP-expressing plasmids were transfected into the cells using polyethylenimine "Max". The cells were washed four times with cold PBS and subsequently lysed in lysis buffer (10 mM Tris-HCl [pH 7.5], 150 mM NaCl, and 1% NP-40) at 4°C at 48 h post-transfection. The supernatant was collected by centrifugation at 16,510 × *g* for 15 min at 4°C. The lysates were incubated with GST-tagged anti-GFP nanobody (87, 88) on a rotator at 4°C overnight. After washing 10 times with cold lysis buffer, the agarose was boiled in SDS sample buffer to extract U65-binding protein. LC-MS was conducted to determine U65-binding proteins.

### ELISA

IFNβ protein concentrations in the culture media were determined using the AuthentiKine Human IFN-beta ELISA Kit (Proteintech) according to the manufacturer’s protocol. Briefly, 293T cells were transfected with the indicated plasmids. At 24 h post-transfection, the cells were further transfected with 0.4 µg of poly(dA:dT). At 18 h post-poly(dA:dT) stimulation, the culture media were collected and the IFNβ protein concentration was evaluated.

### Coimmunoprecipitation

Coimmunoprecipitation was performed as previously described (78) with some modifications. Briefly, 293T cells were transfected with pCAGSS-HA-U65 and pCAGSS-FLAG-H2ACG/H2AC7 or pCAGSS-HA-U65 alone. At 48 h post-transfection, the cells were washed four times with cold PBS and subsequently lysed in lysis buffer at 4°C. The supernatant was collected by centrifugation at 12,000 × *g* for 5 min at 4°C. The lysates were incubated with anti-HA-tag mAb-magnetic agarose on a rotator at 4°C overnight. After washing four times with cold lysis buffer, the agarose was boiled in SDS sample buffer and subjected to SDS-PAGE. The subsequent Western blot was performed as described above.

### Sequence alignment and PTM site prediction

Amino acid sequences of human canonical H2A (UniProt ID: P04908), H2A.X (UniProt ID: P16104), H2ACG (UniProt ID: A0A0U1RR32), H2AC7 (UniProt ID: P20671), and U65 (UniProt ID: Q9QJ19), were retrieved from the UniProt database (https://www.uniprot.org/). Multiple sequence alignment was performed using MAFFT (version 7) with default parameters via the online server at https://mafft.cbrc.jp/alignment/server/ (89). Alignment results were manually inspected, and conserved regions were annotated. Prediction of PTM sites, including phosphorylation, ubiquitination, acetylation, and methylation, was performed using MusiDeep (SVM version) at https://www.musite.net/ (90).

### Virus infection

HHV-6B strain Z29 was propagated in cord blood mononuclear cells, and virus stocks were prepared as previously described (91). 293T-hCD134 cells were transfected with a sh-U65-expressing plasmid, along with the indicated plasmids, and incubated for 24 h. The cells were then incubated with HHV-6B Z29 (MOI = 0.1) at 37°C. After viral absorption for 1 h, the cells were washed with DMEM and then cultured for an additional 3 days. At the indicated time point, the mRNA levels of human IFNβ and viral copies in the infected cells were measured by RT-qPCR and qPCR, respectively.

### IFA

IFA was performed as previously described (86) with some modifications. Briefly, 293T-hCD134 cells (5 × 10^4^) were plated onto chamber slides. The cells were transfected with a sh-U65-expressing plasmid using polyethylenimine "Max". At 24 h post-transfection, the cells were infected with HHV-6B virus (MOI = 0.1) for 1 h, washed with DMEM, then cultured in fresh medium for 3 days. The cells were then fixed in 4% paraformaldehyde for 20 min, washed with PBS, and permeabilized with 0.5% Triton X-100 for 10 min. After washing with PBS, the cells were incubated overnight at room temperature with a mouse antibody against HHV-6 p41 early antigen. At the following day, the cells were washed with PBS and incubated with Alexa Fluor 488-conjugated goat anti-mouse IgG (H + L) F(ab′)2 at room temperature for 1 h. After washing with PBS, the cells were mounted using ProLong Diamond Antifade Mounting with 4′,6-diamidino-2-phenylindole (Thermo Fisher Scientific). The images were captured using the BX60 microscope (Olympus Corporation, Tokyo, Japan). For the U65 localization and the codistribution of U65 with H2ACG/H2AC7 or H2A, transfected 293T cells were fixed, permeabilized, and blocked as described above. The cells were incubated with primary antibodies at 4°C overnight, followed by incubation with Alexa Fluor 488 conjugated goat anti-mouse IgG (H + L) F(ab′)_2_ and Alexa Fluor 555 conjugated goat anti-mouse IgG (H + L) F(ab′)_2_ for 1 h. The images were captured using the BZ-X810 microscope (Keyence, Osaka, Japan) or the LSM780 confocal laser scanning microscope (Carl Zeiss, Jena, Germany).

### Statistical analysis

Statistical significance was evaluated using two-tailed Student’s *t*-test or analysis of variance with Tukey’s post hoc test. A *P* value of less than 0.05 was considered significant.

## Data Availability

Data supporting the findings of this study are presented within the article.
